# Assessment of Risk Factors and Biomarkers Associated With Risk of Cardiovascular Disease Among Women Consuming a Mediterranean Diet

**DOI:** 10.1001/jamanetworkopen.2018.5708

**Published:** 2018-12-07

**Authors:** Shafqat Ahmad, M. Vinayaga Moorthy, Olga V. Demler, Frank B. Hu, Paul M Ridker, Daniel I. Chasman, Samia Mora

**Affiliations:** 1 Department of Medical Sciences, Molecular Epidemiology, Uppsala University, Uppsala, Sweden; 2 Preventive Medicine Division, Brigham and Women’s Hospital, Harvard Medical School, Boston, Massachusetts; 3 Center for Lipid Metabolomics, Brigham and Women’s Hospital, Harvard Medical School, Boston, Massachusetts; 4 Department of Nutrition, Harvard T.H. Chan School of Public Health, Boston, Massachusetts; 5 Cardiovascular Division, Brigham and Women's Hospital, Harvard Medical School, Boston, Massachusetts; 6 Channing Division of Network Medicine, Department of Medicine, Brigham and Women's Hospital and Harvard Medical School, Boston, Massachusetts

## Abstract

**Importance:**

Higher Mediterranean diet (MED) intake has been associated with lower risk of cardiovascular disease (CVD), but limited data are available about the underlying molecular mechanisms of this inverse disease association in human populations.

**Objective:**

To better characterize the relative contribution of traditional and novel factors to the MED-related risk reduction in CVD events in a US population.

**Design, Setting, and Participants:**

Using a prospective cohort design, baseline MED intake was assessed in 25 994 initially healthy US women in the Women’s Health Study who were followed up to 12 years. Potential mediating effects of a panel of 40 biomarkers were evaluated, including lipids, lipoproteins, apolipoproteins, inflammation, glucose metabolism and insulin resistance, branched-chain amino acids, small-molecule metabolites, and clinical factors. Baseline study information and samples were collected between April 30, 1993, and January 24, 1996. Analyses were conducted between August 1, 2017, and October 30, 2018.

**Exposures:**

Intake of MED is a 9-category measure of adherence to a Mediterranean dietary pattern. Participants were categorized into 3 levels based on their adherence to the MED.

**Main Outcomes and Measures:**

Incident CVD confirmed through medical records and the proportion of CVD risk reduction explained by mediators.

**Results:**

Among 25 994 women (mean [SD] age, 54.7 [7.1] years), those with low, middle, and upper MED intakes composed 39.0%, 36.2%, and 24.8% of the study population and experienced 428 (4.2%), 356 (3.8%), and 246 (3.8%) incident CVD events, respectively. Compared with the reference group who had low MED intake, CVD risk reductions were observed for the middle and upper groups, with respective HRs of 0.77 (95% CI, 0.67-0.90) and 0.72 (95% CI, 0.61-0.86) (*P *for trend < .001). The largest mediators of the CVD risk reduction of MED intake were biomarkers of inflammation (accounting for 29.2% of the MED-CVD association), glucose metabolism and insulin resistance (27.9%), and body mass index (27.3%), followed by blood pressure (26.6%), traditional lipids (26.0%), high-density lipoprotein measures (24.0%) or very low-density lipoprotein measures (20.8%), with lesser contributions from low-density lipoproteins (13.0%), branched-chain amino acids (13.6%), apolipoproteins (6.5%), or other small-molecule metabolites (5.8%).

**Conclusions and Relevance:**

In this study, higher MED intake was associated with approximately one-fourth relative risk reduction in CVD events, which could be explained in part by known risk factors, both traditional and novel.

## Key Points

**Question:**

Is the Mediterranean diet (MED) associated with lower risk of cardiovascular disease (CVD) events in a US population, and, if so, what are the underlying mechanisms?

**Findings:**

In this cohort study of 25 994 US women, higher baseline MED intake was associated with up to 28% relative risk reduction in CVD events. For the MED-CVD risk reduction, biomarkers of inflammation, glucose metabolism and insulin resistance, and adiposity contributed most to explaining the association.

**Meaning:**

Higher MED intake was associated with approximately one-fourth relative risk reduction in CVD, which could be explained in part by known risk factors, both traditionally measured and novel ones.

## Teaser

This cohort study examines the association between Mediterranean diet intake and cardiovascular risks and attempts to identify which biological mechanisms underlie the association among participants of the Women’s Health Study.

## Introduction

Modification of overall dietary patterns rather than individual dietary attributes are proposed as a more effective approach for cardiovascular disease (CVD) prevention and intervention.^1^ Based on clinical trial evidence,^2,3^ prospective cohort studies,^4,5,6^ recommendations of the American Heart Association^7^ and the *2015-2020 Dietary Guidelines for Americans*,^8^ a Mediterranean diet (MED) pattern is associated with prevention of CVD, even in non-Mediterranean populations.

Two European randomized clinical trials conducted in Mediterranean countries examined a MED intake intervention vs a control diet and found significant reductions in clinical CVD events. In the Lyon Diet Heart trial, 605 French men with a first myocardial infarction (MI) were randomized to MED intervention vs American Heart Association Step 1 control diet, resulting in 50% to 70% lower relative risk of recurrent CVD.^9^ Most of the measured intermediate CVD biomarkers (including traditional lipids) were similar between the study groups.^10^ Subsequently, the Prevención con Dieta Mediterránea (PREDIMED) trial found that a MED intervention enriched with nuts or extra-virgin olive oil reduced first CVD events by 30% compared with a control low-fat diet in a high-risk Spanish primary prevention population.^2,3^ The MED in this Spanish population at increased risk for CVD was associated with favorable changes in several CVD risk factors, including reduced levels of oxidized low-density lipoproteins (LDL) and triglycerides (TG), increases in high-density lipoproteins (HDL), and improvements in blood pressure, insulin sensitivity, and circulating inflammatory molecules,^11^ but it is unclear which of these favorable changes may mediate the MED benefit on CVD event reduction. No significant weight reduction was observed in either 3 months or 1 year of intervention in the PREDIMED study.^11,12^ Recently, the PREDIMED study’s original findings^3^ have been retracted and republished,^13^ although the republished results are consistent with the previously reported findings for MED benefit.

No randomized trials of MED intervention have been conducted in the United States for clinical outcomes. Observational studies in US populations have reported that a 20-percentile higher MED intake was associated with 9% relative risk reduction in CVD events in short-term follow-up of up to 4 years.^14^ It is uncertain whether MED intake protects against CVD events in US populations over the long term.

Furthermore, the precise mechanisms through which MED intake is associated with reduction in long-term risk of CVD events are not well understood. Therefore, we aimed to (1) examine whether higher MED intake was associated with lower CVD event risk in a US population of initially healthy individuals with long-term (>10 years) follow-up, and (2) better characterize and quantify the relative contribution of a panel of 40 traditional and novel factors to the MED-associated risk reduction in CVD events.

## Methods

### Study Population

Our study followed the Strengthening the Reporting of Observational Studies in Epidemiology (STROBE) reporting guideline for cohort studies. The study population is derived from the Women’s Health Study, as reported previously.^15,16,17^ Briefly, 39 876 female health professionals aged 45 years or older and free from CVD at baseline (1991-1995) were randomized to either to low-dose aspirin or vitamin E. In the Women’s Health Study, 28 345 female participants provided baseline blood samples. For the current analyses, we included 25 994 participants, after excluding participants with missing information for any biomarkers (n = 1614) or MED intake assessed from semiquantitative food-frequency questionnaires (n = 737). To assess the dietary pattern, a validated semiquantitative food-frequency questionnaire including 131 food items was administered to study participants at baseline.^18^ Demographic information was collected from baseline questionnaires asking about history of hypertension, use of postmenopausal hormone therapy, smoking, physical activity, alcohol consumption, and family history of premature MI, among others. Self-reported weight and height were reported, and body mass index (BMI) was calculated. Systolic and diastolic blood pressures were also reported at baseline. All participants provided written informed consent, and the study protocols were approved by the Partners Institutional Review Board, Boston, Massachusetts.

### MED Score

The MED score ranges from 0 to 9, with a higher score representing better adherence to the MED.^19^ Scoring is based on 9 components of MED intake, with 1 point given if intake is above the food-frequency questionnaire’s study median intake for vegetables (excluding potatoes), fruits, nuts, whole grains, legumes, fish, and the ratio of monounsaturated fatty acids to saturated fatty acids; for alcohol intake, 1 point was given if intake ranged from 5 to 15 g/d (otherwise 0 points were given); for red and processed meats, 1 point was given if the intake was below the study-specific median (otherwise 0 points were given). For the current analyses, we categorized the participants according to 3 levels of MED (scores of 0-3, 4-5, and 6-9).

### Ascertainment of Cardiovascular Events

The primary end point for the current analyses was incident CVD,^20^ defined as medical record–confirmed first events of MI, stroke, coronary arterial revascularization, and cardiovascular death. Coronary and stroke events were also examined separately. Because baseline measures of MED intake attenuate with time, we considered our primary analyses with a maximum follow-up of 12 years from baseline, which also corresponded to approximately half the follow-up time. Secondary analyses were performed in the sample with a median (interquartile range) follow-up of 21.4 (19.2-22.1) years.

### Blood Collection and Measurement of Biomarkers

At baseline, blood samples were collected in EDTA tubes, which were centrifuged and stored at −170°C until analyses were performed. Glycated hemoglobin (HbA_1c_) was quantified with an immunoturbidometric assay (Roche Diagnostics). High-sensitivity C-reactive protein (hsCRP) and lipoprotein(a) (Lp[a]) were assayed by turbidimetric immunoassays using the Hitachi-911 analyzer (Roche Diagnostics) with reagents and calibrators from Denka Seiken.^15^ Total cholesterol, HDL cholesterol (HDL-C), and LDL-C were measured enzymatically using tests from Roche Diagnostics and Genzyme. Levels of TG were enzymatically measured (Roche Diagnostics) with correction for endogenous glycerol. Apolipoprotein (apo) B_100_ and apo AI were quantified using turbidometric assays (DiaSorin). Soluble intracellular adhesion molecule 1 (ICAM-1) was measured using enzyme-linked immunosorbent assay (R&D Systems). Fibrinogen was measured using an turbidimetric immunoassay (Kamiya Biomedical). Creatinine was measured using a rate-blanked method based on Jaffe reaction (Roche Diagnostics). Homocysteine was enzymatically measured through the Hitachi-917 analyzer (Roche Diagnostics) using the calibrators and reagents from Catch, Inc.

### Nuclear Magnetic Resonance Spectroscopy Biomarkers

Lipoprotein subfraction particles (concentration and size) for LDL, HDL, and very low-density lipoproteins (VLDL) as well as several circulating small-molecule metabolites were measured by targeted nuclear magnetic resonance (NMR) spectroscopy.^21,22,23^ All NMR spectroscopy measures were performed using H-NMR (400 MHz) LipoProfile-IV (LipoScience [now LabCorp]), including branched-chain amino acids (BCAA [valine, leucine, isoleucine]); citrate^21^; glycoprotein acetylation, which reflects the aggregated level of several glycosylated acute phase proteins and is a measure of inflammation^24^; lipoprotein insulin resistance index and insulin resistance diabetes risk factor index, which are insulin resistance scores and include subfractions of triglyceride-rich lipoproteins, HDL particles, and LDL particles; short-term diabetes risk factor index, which predicts short-term diabetes incidence^25^; and 5-year diabetes risk factor index, which correlates with impaired insulin resistance and insulin secretion.^26^

### Statistical Analysis

Cox regression models were used to compute hazard ratios (HRs) with corresponding 95% confidence intervals using the lowest category (MED score, 0-3) as the reference. Two-sided *P* < .05 was considered as significant. Tests for linear trends were performed using the median value for each of the MED groups. Biomarkers that were not normally distributed (TG, hsCRP, Lp[a], and homocysteine) were log transformed.

To test whether biomarkers satisfied criteria for potential mediators, we followed the steps defined by Baron and Kenny.^27^ We first tested the significance of the association of MED intake with CVD, and then retested the association in a separate model with each potential mediator. Models were adjusted for the confounders age, randomized treatment assignment, and energy intake (Table 1 and Table 2). Next, we evaluated the magnitude of the change in the HRs for the highest vs lowest MED intake group, with and without adjustment for each mediator (Table 3 and Table 4). A larger change in the HR toward the null implies a larger mediating effect of the risk factor on the association between MED intake and reduction in CVD events. Then, on an a priori basis, we grouped together a set of variables that are generally considered to be potential confounders rather than mediators (smoking [never, former, and current], menopausal status, postmenopausal hormone use, exercise, and parental history of MI). We included this set of variables together with age, randomized treatment, and energy intake in a single model, referring to this as the *basic model*. Also on an a priori basis, we grouped other risk factors generally considered to be potential mediators into sets on the basis of their pathophysiological effects. For example, to analyze the combined effects of traditional lipids, we combined total cholesterol, LDL-C, HDL-C, and TG as 1 set. We combined Lp(a), apo AI, and apo B_100_ as 1 set. Fibrinogen, hsCRP, ICAM-1, and glycoprotein acetylation were combined together as 1 set considering their role in inflammation. Diabetes, HbA_1c_, lipoprotein insulin resistance index, insulin resistance diabetes risk factor index, short-term diabetes risk factor index, and 5-year diabetes risk factor index were combined as the glucose metabolism and insulin resistance set. We considered LDL, LDL particle size and concentration, and apo B_100_ the LDL set, while HDL, HDL particle size and concentration, and apo AI were combined as the HDL set. We combined TG-rich lipoprotein subfraction particle concentration, TG-rich lipoprotein particle size, and TG in the VLDL set. Total BCAA, citrate, creatinine, and homocysteine were combined into the small-molecule metabolite group, and total BCAA was also examined separately. Hypertension and systolic and diastolic blood pressures were combined as the hypertension group. Body mass index was examined separately.

**Table 1.** Baseline Characteristics According to MED Intake

Characteristic	MED Score 0-3 (n = 10 140)^a^	MED Score 4-5 (n = 9416)^a^	MED Score ≥6 (n = 6483)^a^	*P* Value for Trend
Age, median (IQR), y	51.9 (48.4-57.4)	53.4 (49.1-59.2)	54.2 (49.8-60.7)	<.001
Current smoking, No. (%)	1594 (15.7)	969 (10.3)	403 (6.3)	<.001
Exercise, No. (%)				
Rarely or never	4564 (45.0)	3357 (35.7)	1681 (26.1)	<.001
<1 Time/wk	2046 (20.2)	1789 (19.0)	1247 (19.4)	
1-3 Times/wk	2716 (26.8)	3156 (33.5)	2467 (38.3)	
≥4 Times/wk	810 (8.0)	1111 (11.8)	1042 (16.2)	
Alcohol consumption, No. (%)				
Rarely	5051 (49.8)	3990 (42.4)	2294 (35.6)	<.001
1-3 Drinks/mo	1478 (14.6)	1254 (13.3)	714 (11.1)	
1-6 Drinks/wk	2775 (27.4)	3144 (33.4)	2556 (39.7)	
≥1 Drinks/d	834 (8.2)	1026 (10.9)	872 (13.6)	
Vegetables, median (IQR), servings/d	2.3 (1.6-3.1)	3.7 (2.8-5.0)	5.2 (4.1-6.8)	<.001
Fruits, median (IQR), servings/d	1.3 (0.8-1.8)	2.1 (1.4-2.9)	2.8 (2.2-3.7)	<.001
Nuts, median (IQR), servings/d	0 (0-0.07)	0.07 (0-0.13)	0.07 (0-0.14)	<.001
Whole grains, median (IQR), servings/d	0.7 (0.3-1.1)	1.2 (0.7-1.9)	1.8 (1.3-2.8)	<.001
Legumes, median (IQR), servings/d	0.2 (0.1-0.4)	0.4 (0.2-0.6)	0.6 (0.4-0.9)	<.001
Fish, median (IQR), servings/d	0.1 (0.1-0.2)	0.2 (0.1-0.3)	0.3 (0.2-0.5)	<.001
Ratio of monounsaturated to saturated fat, median (IQR)	1.1 (1.0-1.1)	1.1 (1.0-1.2)	1.2 (1.1-1.3)	<.001
Red meat, median (IQR), servings/d	0.6 (0.4-1.0)	0.6 (0.3-1.0)	0.5 (0.3-0.9)	<.001
Processed meats, median (IQR), servings/d	0.1 (0.1-0.3)	0.1 (0-0.2)	0.1 (0-0.2)	<.001
Hypertension, No. (%)	2540 (25.1)	2324 (24.7)	1591 (24.7)	.80
Diabetes, No. (%)	231 (2.3)	201 (2.1)	160 (2.5)	.35
Postmenopausal, No. (%)	5075 (50.1)	5220 (55.5)	3798 (59.1)	<.001
Body mass index, median (IQR)^b^	25.0 (22.6-28.8)	24.9 (22.5-28.3)	24.3 (22.1-27.5)	<.001
Parental history of myocardial infarction <60 y, No. (%)	1461 (14.4)	1313 (13.9)	864 (13.4)	.20

Abbreviations: IQR, interquartile range; MED, Mediterranean diet.

^a^ The MED score is based on 9 components of MED intake. A higher score represents better adherence to the MED, ranging from 0 to 9. For the current analysis, we categorized the participants according to 3 levels of MED (group 1 [lowest]: MED score 0-3; group 2: MED score 4-5; and group 3 [highest]: MED score 6-9). *P* values apply across 3 levels of MED.

^b^ Calculated as weight in kilograms divided by height in meters squared.

**Table 2.** Baseline Biomarker Levels According to MED Intake

Biomarker	Median (IQR)^a^			*P* Value for Trend
	MED Score 0-3	MED Score 4-5	MED Score ≥6	
Blood pressure, mm Hg				
Systolic	125.0 (115.0-135.0)	125.0 (115.0-135.0)	125.0 (115.0-125.0)	.82
Diastolic	80.0 (70.0-80.0)	80.0 (70.0-80.0)	80.0 (70.0-80.0)	.02
Lipids, cholesterol, mg/dL				
LDL	121.8 (100.9-144.4)	121.3 (101.1-144.2)	121.0 (100.4-144.9)	.83
HDL	51.2 (42.7-61.4)	52.5 (43.7-62.9)	53.6 (44.6-64.3)	<.001
Triglycerides	118.0 (84.0-173.0)	118.0 (83.0-174.0)	117.0 (83.0-169.0)	.001
Total	207.0 (183.0-234.0)	208.0 (184.0-235.0)	209.0 (184.0-236.0)	.03
Lipoproteins, mg/dL				
Lipoprotein(a)	10.4 (4.3-32.1)	10.8 (4.6-33.3)	10.8 (4.4-33.1)	.04
Apolipoprotein AI	147.7 (131.1-166.2)	149.7 (133.3-168.4)	151.8 (134.9-170.7)	<.001
Apolipoprotein B_100_	100.2 (84.1-120.9)	99.7 (83.4-120.5)	99.7 (83.9-120.7)	.87
LDL particles and size				
LDL particle concentration, nmol/L	1566.0 (1330.0-1840.0)	1567.0 (1330.0-1838.0)	1569.0 (1327.0-1835.0)	.99
LDL particle size, nm	20.9 (20.6-21.2)	20.9 (20.6-21.2)	20.9 (20.6-21.2)	.003
HDL particles and size				
HDL particle concentration, μmol/L	24.2 (21.8-26.8)	24.4 (22.1-27.1)	24.6 (22.2-27.2)	<.001
HDL particle size, nm	8.9 (8.6-9.2)	8.9 (8.7-9.2)	8.9 (8.7-9.2)	<.001
VLDL measures				
TRL particle concentration, nmol/L	167.2 (132.2-208.6)	166.7 (129.7-208.9)	165.3 (129.2-207.4)	.09
TRL particle size, nm	42.5 (38.6-48.1)	42.6 (38.6-47.9)	42.4 (38.5-47.6)	.02
Glycemic				
Hemoglobin A_1c_, % of total hemoglobin	5.00 (4.8-5.2)	4.99 (4.8-5.2)	5.00 (4.8-5.2)	.25
Glucose metabolism and insulin resistance^b^				
Lipoprotein insulin resistance index score	41.0 (21.0-62.0)	40.0 (20.0-61.0)	38.0 (20.0-58.0)	<.001
Insulin resistance diabetes risk factor score	33.0 (15.0-54.0)	32.0 (15.0-52.0)	29.0 (13.0-49.0)	<.001
Short-term diabetes risk factor index score	49.0 (42.0-53.0)	49.0 (40.0-53.0)	48.0 (40.0-52.0)	<.001
5-y diabetes risk factor index score	46.0 (31.0-63.0)	45.0 (29.0-61.0)	42.0 (28.0-59.0)	<.001
Inflammation				
High-sensitivity C-reactive protein, mg/L	2.1 (0.8-4.5)	2.0 (0.8-4.3)	1.8 (0.8-4.0)	<.001
Fibrinogen, mg/dL	351.4 (307.3-405.0)	350.8 (308.3-401.5)	347.4 (305.2-398.4)	.001
Soluble intercellular adhesion molecule 1, ng/mL	345.6 (302.2 (400.2)	342.1 (300.7-393.1)	337.8 (297.7-384.8)	<.001
Glycoprotein acetylation, μmol/L	385.0 (340.0-433.0)	382.0 (339.0-429.0)	379.0 (335.0-424.0)	<.001
Branched-chain amino acids, μmol/L				
Total branched-chain amino acids	404.0 (351.0-465.0)	400.0 (349.0-460.0)	397.0 (348.0-455.0)	<.001
Valine	221.0 (193.0-253.0)	220.0 (193.0-250.0)	218.0 (192.0-248.0)	.004
Leucine	132.0 (111.0-156.0)	131.0 (110.0-154.0)	131.0 (110.0-154.0)	.003
Isoleucine	51.0 (40.0-65.0)	50.0 (38.0-63.0)	49.0 (38.0-62.0)	<.001
Small-molecule metabolites				
Citrate, μmol/L	94.0 (79.0-110.0)	94.0 (79.0-111.0)	93.0 (78.0-110.0)	.05
Creatinine, mg/dL	0.7 (0.6-0.8)	0.7 (0.6-0.8)	0.7 (0.6-0.8)	.43
Homocysteine, μmol/L	10.7 (8.8-13.2)	10.3 (8.6-12.7)	10.2 (8.6 (12.4)	<.001

Abbreviations: HDL, high-density lipoprotein; IQR, interquartile range; LDL, low-density lipoprotein; MED, Mediterranean diet; TRL, triglyceride-rich lipoprotein; VLDL, very low-density lipoprotein.

SI conversion factors: To convert HDL and LDL cholesterol to mmol/L, multiply by 0.0253; triglycerides to mmol/L, multiply by 0.0113; lipoprotein(a) to μmol/L, multiply by 0.0357; apolipoprotein AI and Apolipoprotein B_100_ to g/L, multiply by 0.01; hemoglobin A_1c_ to proportion of total hemoglobin, multiply by 0.01; C-reactive protein to nmol/L, multiply by 9.524; fibrinogen to μmol/L, multiply by 0.0294; and creatinine to μmol/L, multiply by 88.4.

^a^ We categorized the participants according to 3 levels of MED (scores of 0-3, 4-5, and 6-9).

^b^ Five-year diabetes risk factor index, insulin resistance diabetes risk factor, short-term diabetes risk factor index, and lipoprotein insulin resistance index are scored on a scale of 1 to 100, with higher numbers indicating higher risk.

**Table 3.** Association of MED Intake With Cardiovascular Disease Events (12-y Follow-up) After Adjustment for Cardiovascular Disease Risk Factors

Biomarker	HR (95% CI)^a^			*P* Value for Trend
	MED Score 0-3	MED Score 4-5	MED Score ≥6	
Age, treatment, and energy–adjusted model	1 [Reference]	0.77 (0.67-0.90)	0.72 (0.61-0.86)	<.001
Age, treatment, and energy–adjusted model plus each of the following added 1 at a time				
Smoking	1 [Reference]	0.83 (0.72-0.96)	0.81 (0.68-0.96)	.009
Alcohol consumption	1 [Reference]	0.79 (0.68-0.91)	0.75 (0.63-0.89)	<.001
Blood pressure				
Hypertension	1 [Reference]	0.79 (0.69-0.92)	0.75 (0.63-0.89)	.001
Systolic, mm Hg	1 [Reference]	0.81 (0.70-0.94)	0.77 (0.65-0.91)	.002
Diastolic, mm Hg	1 [Reference]	0.79 (0.69-0.92)	0.75 (0.63-0.89)	<.001
Traditional lipids, cholesterol, mg/dL				
LDL	1 [Reference]	0.78 (0.67-0.90)	0.72 (0.61-0.86)	<.001
HDL	1 [Reference]	0.80 (0.70-0.93)	0.78 (0.65-0.92)	.002
Triglycerides	1 [Reference]	0.78 (0.68-0.91)	0.75 (0.63-0.89)	<.001
Total	1 [Reference]	0.77 (0.67-0.90)	0.72 (0.60-0.85)	<.001
Apolipoproteins, mg/dL				
Lipoprotein(a)	1 [Reference]	0.77 (0.67-0.89)	0.72 (0.61-0.85)	<.001
Apolipoprotein AI	1 [Reference]	0.79 (0.68-0.91)	0.75 (0.63-0.89)	.001
Apolipoprotein B_100_	1 [Reference]	0.79 (0.69-0.91)	0.75 (0.64-0.87)	<.001
LDL particles and size				
LDL particle concentration, nmol/L	1 [Reference]	0.78 (0.68-0.90)	0.73 (0.62-0.87)	<.001
LDL particle size, nm	1 [Reference]	0.78 (0.67-0.90)	0.74 (0.62-0.88)	<.001
HDL particles and size				
HDL particle concentration, μmol/L	1 [Reference]	0.78 (0.67-0.90)	0.73 (0.61-0.87)	<.001
HDL particle size, nm	1 [Reference]	0.80 (0.69-0.92)	0.76 (0.64-0.91)	.001
VLDL particles and size				
TRL particle concentration, nmol/L	1 [Reference]	0.78 (0.67-0.90)	0.74 (0.62-0.88)	<.001
TRL particle size, nm	1 [Reference]	0.78 (0.68-0.91)	0.74 (0.62-0.88)	<.001
Glucose metabolism and insulin resistance				
Diabetes	1 [Reference]	0.79 (0.69-0.92)	0.72 (0.61-0.86)	.002
Hemoglobin A_1c_, % of total hemoglobin	1 [Reference]	0.77 (0.67-0.89)	0.74 (0.62-0.88)	<.001
Lipoprotein insulin resistance index score	1 [Reference]	0.80 (0.69-0.92)	0.77 (0.65-0.92)	.002
Insulin resistance diabetes risk factor score	1 [Reference]	0.81 (0.70-0.94)	0.79 (0.67-0.94)	.005
Short-term diabetes risk factor index score	1 [Reference]	0.79 (0.68-0.91)	0.75 (0.63-0.89)	<.001
5-y diabetes risk factor index score	1 [Reference]	0.81 (0.70-0.94)	0.80 (0.68-0.95)	.007
Inflammation pathway				
High-sensitivity C-reactive protein, mg/L	1 [Reference]	0.79 (0.69-0.92)	0.76 (0.64-0.91)	.001
Fibrinogen, mg/dL	1 [Reference]	0.79 (0.68-0.91)	0.74 (0.63-0.88)	<.001
Soluble intercellular adhesion molecule 1, ng/mL	1 [Reference]	0.81 (0.70-0.93)	0.77 (0.65-0.92)	.002
Glycoprotein acetylation , μmol/L	1 [Reference]	0.79 (0.68-0.92)	0.76 (0.64-0.90)	<.001
Branched-chain amino acids, μmol/L				
Total branched-chain amino acids	1 [Reference]	0.79 (0.68-0.91)	0.74 (0.63-0.88)	<.001
Valine	1 [Reference]	0.78 (0.68-0.91)	0.74 (0.62-0.88)	<.001
Leucine	1 [Reference]	0.78 (0.67-0.90)	0.73 (0.62-0.87)	<.001
Isoleucine	1 [Reference]	0.80 (0.69-0.92)	0.75 (0.63-0.89)	.001
Small-molecule metabolites				
Citrate, μmol/L	1 [Reference]	0.77 (0.67-0.90)	0.72 (0.61-0.86)	<.001
Creatinine, mg/dL	1 [Reference]	0.77 (0.67-0.90)	0.72 (0.61-0.86)	<.001
Homocysteine, μmol/L	1 [Reference]	0.79 (0.68-0.91)	0.74 (0.62-0.88)	<.001

Abbreviations: HDL, high-density lipoprotein; HR, hazard ratio; LDL, low-density lipoprotein; MED, Mediterranean diet; TRL, triglyceride-rich lipoprotein; VLDL, very low-density lipoprotein.

SI conversion factors: To convert HDL and LDL cholesterol to mmol/L, multiply by 0.0253; triglycerides to mmol/L, multiply by 0.0113; lipoprotein(a) to μmol/L, multiply by 0.0357; apolipoprotein AI and Apolipoprotein B_100_ to g/L, multiply by 0.01; hemoglobin A_1c_ to proportion of total hemoglobin, multiply by 0.01; C-reactive protein to nmol/L, multiply by 9.524; fibrinogen to μmol/L, multiply by 0.0294; and creatinine to μmol/L, multiply by 88.4.

^a^ We categorized the participants according to 3 levels of MED (scores of 0-3, 4-5, and 6-9). *P* values across 3 levels of MED were all less than .05.

**Table 4.** Association of MED Intake With Cardiovascular Disease Events After Adjustment for Sets of Potential Mediators

Model Adjustment	HR (95% CI)^a^			*P* Value for Trend
	MED Score 0-3	MED Score 4-5	MED Score ≥6	
Age, treatment, and energy–adjusted model	1 [Reference]	0.77 (0.67-0.90)	0.72 (0.61-0.86)	<.001
Basic model^b^	1 [Reference]	0.85 (0.73-0.98)	0.85 (0.71-1.01)	.04
Basic model plus each set of risk factors below, added 1 group at a time^c^				
Hypertension: history of hypertension, systolic and diastolic blood pressure	1 [Reference]	0.88 (0.76-1.02)	0.89 (0.74-1.06)	.14
Body mass index	1 [Reference]	0.87 (0.75-1.01)	0.89 (0.75-1.06)	.14
Traditional lipids: total, LDL, HDL cholesterol, triglycerides	1 [Reference]	0.87 (0.75-1.00)	0.89 (0.74-1.06)	.13
Apolipoproteins: lipoprotein(a), apolipoprotein AI, apolipoprotein B_100_	1 [Reference]	0.86 (0.74-0.99)	0.86 (0.72-1.02)	.06
LDL measures: LDL particle size and concentration, LDL cholesterol, apolipoprotein B_100_	1 [Reference]	0.86 (0.74-1.00)	0.87 (0.73-1.03)	.08
HDL measure: HDL particle size and concentration, HDL cholesterol, apolipoprotein AI	1 [Reference]	0.87 (0.75-1.01)	0.88 (0.74-1.05)	.12
VLDL measures: triglyceride-rich lipoprotein particle size and concentrations, triglycerides	1 [Reference]	0.86 (0.75-1.00)	0.88 (0.74-1.05)	.11
Inflammation: hsCRP, fibrinogen, sICAM-1, glycoprotein acetylation	1 [Reference]	0.87 (0.75-1.01)	0.89 (0.75-1.06)	.15
Glucose metabolism and insulin resistance: diabetes, hemoglobin A1c, LPIR, IRDRF, SDRF, DRF5	1 [Reference]	0.88 (0.76-1.02)	0.89 (0.75-1.06)	.15
Branched-chain amino acids	1 [Reference]	0.86 (0.74-1.00)	0.87 (0.73-1.03)	.08
Small-molecule metabolites: citrate, creatinine, homocysteine	1 [Reference]	0.85 (0.74-0.99)	0.86 (0.72-1.02)	.06

Abbreviations: DRF5, 5-year diabetes risk factor index; HDL, high-density lipoprotein; HR, hazard ratio; hsCRP, high sensitivity C-reactive protein; IRDRF, insulin resistance diabetes risk factor; LDL, low-density lipoprotein; LPIR, lipoprotein insulin resistance index; SDRF, short-term diabetes risk factor index; sICAM-1, soluble intercellular adhesion molecule 1; VLDL, very low-density lipoproteins.

^a^ We categorized the participants according to 3 levels of MED (scores of 0-3, 4-5 and ≥6). *P* values across 3 levels of MED were all less than .05.

^b^ Basic model included age, randomized treatment assignment, energy intake, smoking, menopausal status, postmenopausal hormone use, physical activity, and parental history of myocardial infarction before age 60 years. Participants were followed up to 12 years.

^c^ Models were adjusted for the variables in the basic model plus each of the sets of risk factors added 1 group at a time to separate models.

To examine the extent to which each set of risk factors potentially mediated the association of MED intake on incident CVD, we next added these sets, 1 set at a time, to the basic model and examined the magnitude of change in the HRs for the group with the highest MED intake compared with the lowest intake without adjustment (basic model) and with adjustment for each set (adjusted model = basic model + mediator set). The proportion of CVD risk reduction explained by each set of mediators was calculated through the formula (HR_basic_ model − HR_adjusted model_)/(HR_basic model_ − 1) × 100%.^28,29^ As these biomarkers are correlated, their separate contributions can add up to more than 100%. Sensitivity analyses were performed using a counterfactual-framework approach^30^ through use of the SAS PROC CAUSALMED procedure. The results of both mediation approaches were compared for single mediators using MED as a continuous variable.

Sensitivity analyses were also repeated for the separate end points of coronary heart disease (CHD) and total stroke.

## Results

### Baseline Characteristics

In the study sample of 25 994 female participants (mean [SD] baseline age, 54.7 [7.1] years), a total of 1030 participants (3.96%) experienced a first CVD event. Participants with low (MED score ≤3), middle (MED score 4-5), and upper (MED score 6-9) dietary MED intake composed 39.0% (10 140), 36.2% (9416), and 24.8% (6438) of the study population and experienced 428 (4.2%), 356 (3.8%), and 246 (3.8%) incident CVD events, respectively. The risk of CVD events decreased with higher baseline MED intake. Compared with the reference group of low MED intake, CVD risk reductions were observed for the middle and upper groups, with respective HRs of 0.77 (95% CI, 0.67-0.90) and 0.72 (95% CI, 0.61-0.86) (*P *for trend *<* .001). Women with higher MED intake had a higher intake of vegetables, fruits, nuts, whole grains, legumes, and fish; greater ratio of monosaturated to saturated fat; and lower intake of processed and red meat (Table 1). Higher MED intake was generally associated with more favorable profiles of CVD risk factors and biomarkers (Table 2) with some exceptions. For example, total cholesterol was significantly higher with higher MED intake (median [interquartile range], 209.0 [184.0-236.0]) than in the lower MED intake group (median [interquartile range], 207.0 [183.0-234.0]) (*P* = .03), while systolic blood pressure, LDL-C, apo B_100_, LDL particle concentration, creatinine, and HbA_1c_ were similar across the groups (*P* > .05).

### Mediation

All factors except 6 met the criteria of Baron and Kenny^27^ for mediation (Table 1 and Table 2): systolic blood pressure (*P* = .82), LDL-C (*P* = .83), apo B_100_ (*P* = .87), LDL particle concentration (*P* = .99), HbA_1c_ (*P* = .25), and creatinine (*P* = .43) (see Table 2 for median [interquartile range] values with MED scores of 0-3, 4-5, and ≥6). However, significant inverse association of these 6 parameters with MED intake have been reported previously.^31,32,33^ Therefore, we also evaluated the degree of the mediation association of all factors on the association of MED with the outcomes (Table 3 and Table 4).

### MED and Risk of CVD

During a maximum follow-up of 12 years (mean [SD], 11.6 [1.5] years), a total of 1030 individuals experienced a first event, including 681 coronary events and 339 strokes. Compared with the reference group of participants with low MED intake, CVD risk reductions were observed for the middle and upper groups, with respective HRs of 0.77 (95% CI, 0.67-0.90) and 0.72 (95% CI, 0.61-0.86) (*P* for trend < .001) (Table 3). Using the group with MED scores from 0 to 3 as the reference, we observed CVD relative risk reductions of 23% and 28% for groups with scores of 4 to 5 and 6 or greater, respectively, adjusting for age, randomized treatment, and energy intake (Table 3). In separate Cox models that were additionally adjusted with each of the individual biomarkers, 1 at a time, we observed some attenuation of HRs (comparing higher vs lower MED intake) before and after adjustment for most variables except for LDL-C, total cholesterol, Lp(a), citrate, and creatinine (Table 3).

### Adjustment for Each Set of Intermediate Biomarkers on the MED Intake–CVD Association

Next, to determine the extent to which the reduced risk of CVD associated with MED was influenced by potential mediators representing various physiological pathways, each set of mediators was added, 1 set at a time, to the basic model (Table 4). The addition of the hypertension group resulted in an attenuation of the inverse relation, which became nonsignificant (*P* for trend = .14). Similar results were observed in separate models that adjusted for BMI (*P* for trend = .14), traditional lipids (*P* for trend = .13), inflammation (*P* for trend = .15), glucose metabolism and insulin resistance (*P* for trend = .15), LDL measures (*P* for trend = .08), HDL measures (*P* for trend = .12), VLDL measures (*P* for trend = .11), and BCAA (*P* for trend = .08). The addition of apolipoproteins (*P* for trend = .06) and small-molecule metabolites (*P* for trend = .06) resulted in smaller attenuation compared with other lipid-related biomarkers (see Table 4 for HR [95% CI] values with MED scores of 4-5 and ≥6 vs MED scores of 0-3).

For the separate end points of stroke and CHD, MED intake had stronger inverse association with CHD compared with stroke (eTable 1 in the Supplement), although generally similar patterns were observed.

### Proportion of Reduction of CVD Events Explained by Potential Mediators

We estimated the proportion of MED intake reduction in CVD (Figure) that was explained by each set of potential mediators. Inflammation biomarkers made the largest contribution (accounting for 29.2% of the MED-CVD benefit), followed by biomarkers of glucose metabolism and insulin resistance (27.9%), BMI (27.3%), blood pressure (26.6%), traditional lipids (26.0%), and measures of HDL (24.0%) or VLDL (20.8%) metabolism, and, to a lesser extent, LDL measures (13.0%), BCAAs (13.6%), apolipoproteins (6.5%) and small-molecule metabolites (5.8%). We also performed mediation analyses combining all these biomarkers (which are intercorrelated) in 1 model. Compared with the low–MED intake reference group, fully adjusted CVD HRs were 0.88 (95% CI, 0.76-1.02) and 0.89 (95% CI, 0.74-1.06) for the middle- and upper-intake groups, respectively (*P* for trend = .15), with a total mediation effect of 27.3%.

**Figure.**
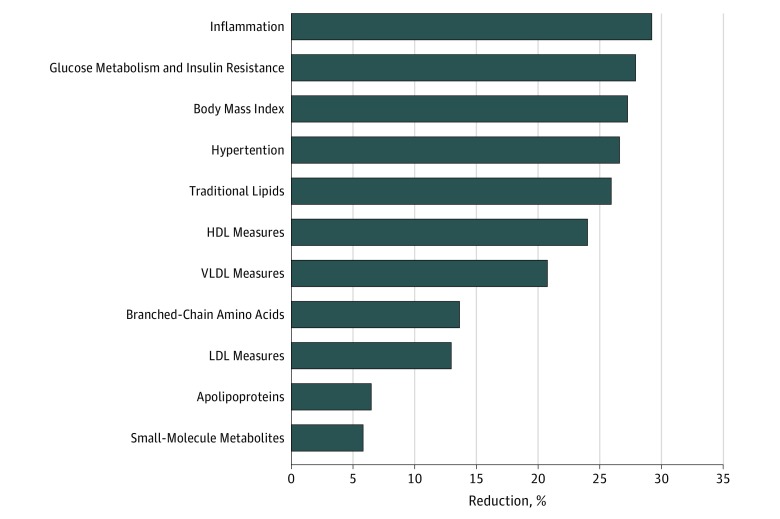
Percentage Reduction in Cardiovascular Disease Events Associated With Mediterranean Diet Explained by Potential Risk Mediators The proportion of the risk reduction for a Mediterranean diet score of 6 or higher (vs the reference group of Mediterranean diet score 0-3) is shown for potential mediators. The percentage mediation effect was calculated through the following formula: (HR_basic model_ − HR _adjusted model_)/ (HR _basic model_ − 1) × 100. The proportions were calculated based on 3 digits, so they might slightly differ from the estimates reported in Table 4. HDL indicates high-density lipoprotein; HR, hazard ratio; LDL, low-density lipoprotein; and VLDL, very low-density lipoprotein.

A generally similar pattern of risk reduction was observed for CHD and stroke risk (eTable 1 and eFigure in the Supplement).

We repeated these analyses using the total follow-up of 21.4 median years and observed materially similar results (eTable 2 in the Supplement). We also compared the reported mediation approach results with the counterfactual framework for single mediators, and the results were similar (eTable 3 in the Supplement).

## Discussion

Although MED intake has been associated with the reduced risk of CVD in observational and interventional studies, it is unclear whether MED intake is associated with long-term CVD benefit in US populations, and what would be the underlying biological mechanisms that may mediate this benefit. In the current study conducted in a large initially healthy population of US women, we observed that higher MED intake was associated with approximately one-quarter lower risk of CVD events over a 12-year follow-up period. Furthermore, the benefit could be explained in part by known risk factors, both traditional and novel. Inflammation explained the largest proportion (29.2%) of the reduction in CVD, followed by glucose metabolism and insulin resistance (27.9%), BMI (27.3%), blood pressure (26.6%), traditional lipids (26.0%), and measures of HDL (24.0%) and VLDL (20.8%) metabolism, with lesser contributions from LDL, BCAAs, or other biomarkers. In total, these results show that these risk factors explained part of the association, but additional unmeasured factors may also contribute.

Previous studies on intermediate outcomes (but not clinical events) have demonstrated favorable effects of adherence to MED intake on cardiometabolic biomarkers including metabolic syndrome,^34^ improved insulin resistance,^35^ lower hsCRP and interleukin-6,^36^ and glucose metabolism.^37^ The PREDIMED trial showed that clinical CVD events may be reduced by approximately 30%,^3^ but the underlying mechanisms related to the protective association of MED intake with CVD is not well defined. Our findings support the role of MED on modifying inflammatory biomarkers, as we estimated that 29.2% of the MED benefit was related to inflammation. A recent study reported that hydroxytyrosol (found in fruits, nuts, legumes, and extra-virgin olive oil) repairs CVD-related oxidative damage and improves blood lipids.^38^ Likewise, in a substudy of 778 participants in PREDIMED, MED intake was associated with improvements in cellular and circulating anti-inflammatory properties.^39^ In a 2-year follow up of randomized treatment intervention with MED, Esposito et al^36^ reported significant improvements in hsCRP and endothelial function compared with the control diet. Dai et al^40^ also reported that reduced inflammation is an important mechanism linking the intake of MED and lower CVD risk.

We also observed the attenuation of the association of MED intake on cardiovascular risk through pathways related to glucose metabolism and insulin resistance, adiposity, blood pressure, traditional lipids, HDL and VLDL measures, and, to a lesser extent, LDL size and particles, but not LDL or total cholesterol. Similarly, Park et al^41^ reported that obesity mediates the association between MED intake on insulin resistance and inflammation biomarkers. It has been reported that components of MED, including peanuts and walnuts, can reduce lipids.^42^ We also observed that 24.0% of the association between MED and reduced CVD was explained by HDL measures. Similarly, in the subsample of the PREDIMED study, MED adherence was associated with increased HDL ability to esterify cholesterol and HDL vasodilatory capacity.^43^

The beneficial association with MED intake was stronger for CVD and CHD than for total stroke in the current study, but the relative contribution of different mediator groups was comparable. A recent meta-analysis also observed weaker or null association between MED intake and stroke.^44^

The MED score is based on the published literature and our a priori hypothesis. A diet score involves some level of arbitrary cut points in terms of which components it contains and the assignment of scores to different levels of intake. Although the MED score that we used in the current study is very similar to what has been reported in the literature about the choice of food items, there are some differences. The score used by Trichopoulou et al^45^ and Pitsavos et al^46^ also included potato intake, and Trichopoulou et al^45^ also included dairy products.

The current study has several advantages, including its prospective design, large sample size, detailed information about MED intake and measured biomarkers that range from conventional to novel risk pathways, and the long follow-up.

### Limitations

There are several limitations that need to be acknowledged. We cannot rule out the possibility of residual confounding related to unmeasured CVD factors. Dietary intake was assessed through food frequency questionnaires, as self-reported diet intake might lead to exposure misclassification, including underreporting and overreporting, although that would attenuate the MED-CVD association toward the null. It is possible that some of the covariates, including hypertension, may be influenced by MED intake, which suggests that these variables could be confounders or mediators. Study participants were US female health care professionals who might have different behaviors than men or higher-risk individuals.

## Conclusions

Our results suggest that a proportion of the lower risk of CVD events with MED intake may be accounted for by known factors related to inflammation, glucose metabolism and insulin resistance, BMI, blood pressure, and lipids (in particular HDL and VLDL). Despite this, a sizeable proportion of the potential benefit of MED intake with CVD risk reduction remains unexplained and requires future investigation into additional mechanisms.

## Notes

Supplement. **eFigure.** Percentage Reduction in CHD and Stroke Events Associated With Mediterranean Diet That Is Explained by Potential Risk Mediators **eTable 1.** Association of Mediterranean Diet With CHD and Stroke Events After Adjustment for Sets of Mediators **eTable 2.** Association of Mediterranean Diet With CVD Events After Adjustment for Sets of Potential Mediators Within Total-Years of Follow-up (Median 21.4 Years) **eTable 3.** Mediation Effect Explained Through Different Risk Factors Regarding Mediterranean Diet Intake With CVD Events
