# Supplementary material for: Assessment of Risk Factors and Biomarkers Associated With Risk of Cardiovascular Disease Among Women Consuming a Mediterranean Diet
Source: JAMA Netw Open. 2018 Dec 7;1(8):e185708. doi: 10.1001/jamanetworkopen.2018.5708 (PMC6324327; doi:10.1001/jamanetworkopen.2018.5708)
Supplement: Supplement. — eFigure. Percentage Reduction in CHD and Stroke Events Associated With Mediterranean Diet That Is Explained by Potential Risk Mediators eTable 1. Association of Mediterranean Diet With CHD and Stroke Events After Adjustment for Sets of Mediators eTable 2. Association of Mediterranean Diet With CVD Events After Adjustment for Sets of Potential Mediators Within Total-Years of Follow-up (Median 21.4 Years) eTable 3. Mediation Effect Explained Through Different Risk Factors Regarding Mediterranean Diet Intake With CVD Events [file jamanetwopen-1-e185708-s001.pdf]

Supplementary Online Content

Ahmad S, Moorthy MV, Demler OV, et al. Assessment of risk factors and biomarkers associated with risk of cardiovascular disease among women consuming a Mediterranean diet. *JAMA Netw Open*. 2018;1(8):e185708. doi:10.1001/jamanetworkopen.2018.5708

eFigure. Percentage Reduction in CHD and Stroke Events Associated With Mediterranean Diet That Is Explained by Potential Risk Mediators

eTable 1. Association of Mediterranean Diet With CHD and Stroke Events After Adjustment for Sets of Mediators

eTable 2. Association of Mediterranean Diet With CVD Events After Adjustment for Sets of Potential Mediators Within Total-Years of Follow-up (Median 21.4 Years)

eTable 3. Mediation Effect Explained Through Different Risk Factors Regarding Mediterranean Diet Intake With CVD Events

This supplementary material has been provided by the authors to give readers additional information about their work.

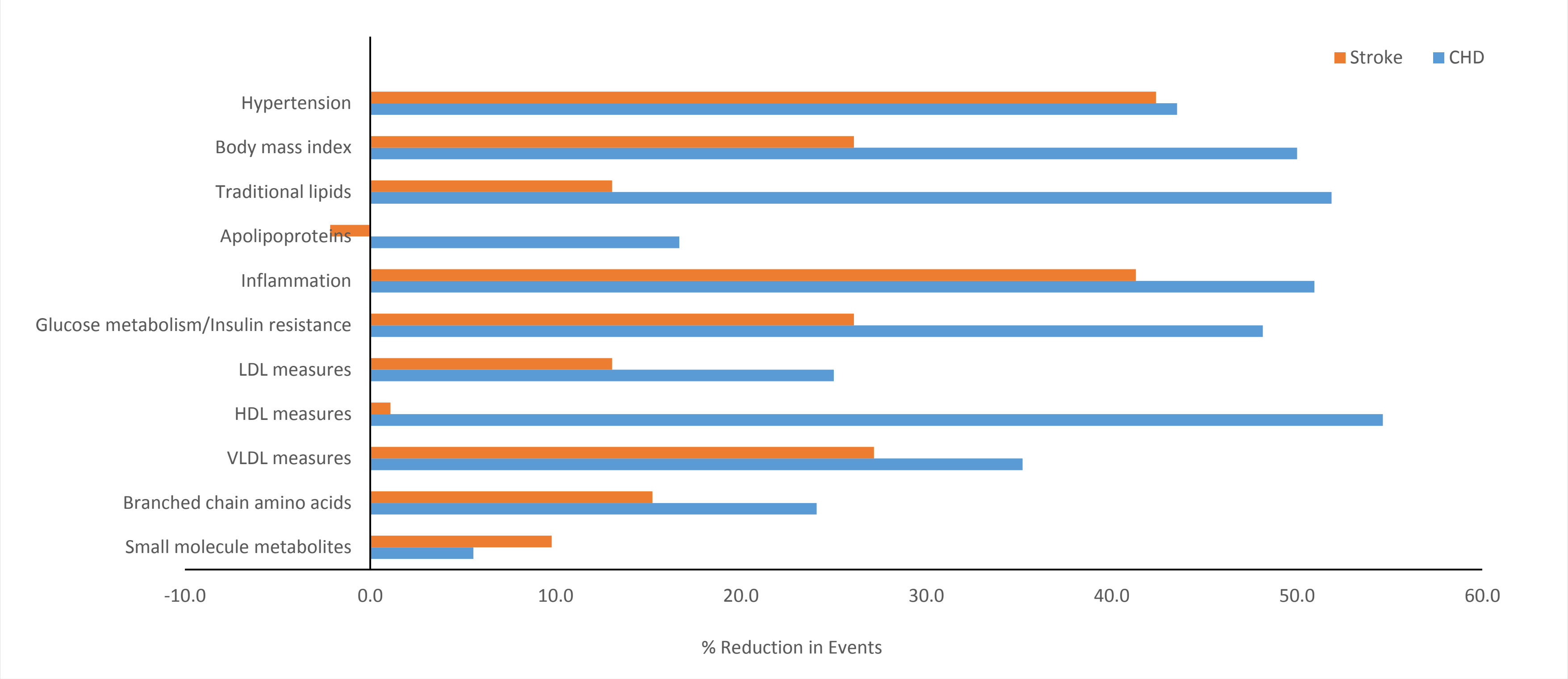

eFigure. Percentage reduction in CHD and stroke events associated with Mediterranean diet that is explained by potential risk mediators. The proportion of the risk reduction for MED ≥ 6 of Mediterranean diet intake (versus the reference group of MED 0-3). The percentage mediation effect was calculated through the following formula: $(HR_{\text{basic model}} - HR_{\text{adjusted model}})/(HR_{\text{basic model}} - 1) * 100$. The proportion were calculated based upon 3 digits so might slightly differ from the estimates reported in eTable 1. HDL measures, High density lipoprotein measures; VLDL, very low density lipoprotein measures; LDL, low density lipoprotein measures.

© 2018 Ahmad S et al. *JAMA Network Open.*

eTable 1. Association of Mediterranean Diet with CHD and Stroke Events after adjustment for sets of mediators				
	Mediterranean Diet			
Biomarkers	MED 0-3	MED 4-5	MED ≥ 6	P for Trend
**Stroke**				
Age, treatment and energy-adjusted model	1 [Reference]	0.82 (0.64-1.06)	0.79 (0.58-1.06)	0.09
Basic model*	1 [Reference]	0.89 (0.69-1.15)	0.91 (0.67-1.24)	0.49
Basic model plus each set of risk factors below, added 1 group at a time†				
Hypertension: History of hypertension, systolic and diastolic blood pressure	1 [Reference]	0.92 (0.71-1.19)	0.95 (0.69-1.29)	0.68
Body mass index	1 [Reference]	0.89 (0.69-1.15)	0.93 (0.68-1.27)	0.59
Traditional lipids: Total, LDL[a], HDL[b] cholesterol, triglycerides	1 [Reference]	0.90 (0.69-1.16)	0.92 (0.68-1.26)	0.54
Apolipoproteins: Lipoprotein(a), Apolipoprotein A1, Apolipoprotein B_{100}	1 [Reference]	0.89 (0.69-1.15)	0.91 (0.67-1.23)	0.48
LDL measures: LDL particle size and concentration, LDL cholesterol, $apoB_{100}$	1 [Reference]	0.90 (0.69-1.16)	0.92 (0.68-1.25)	0.56
HDL measure: HDL particle size and concentration, HDL cholesterol, Apolipoprotein A1	1 [Reference]	0.89 (0.69-1.15)	0.91 (0.67-1.24)	0.49
VLDL[c] measures: Triglyceride-rich lipoprotein particle size and concentrations, triglycerides	1 [Reference]	0.90 (0.70-1.17)	0.93 (0.68-1.27)	0.60
Glucose metabolism/insulin resistances: Diabetes, hemoglobin A1c, LPIR[d], IRDRF[e], SDRF[f], DRF5[g]	1 [Reference]	0.90 (0.70-1.17)	0.93 (0.68-1.27)	0.60
Inflammation: hsCRP[h], fibrinogen, ICAM-1[i], GlycA	1 [Reference]	0.91 (0.70-1.17)	0.95 (0.69-1.29)	0.66
Branched-chain amino acids: total BCAA[j]	1 [Reference]	0.90 (0.69-1.16)	0.92 (0.68-1.26)	0.55
Small molecule metabolites: Citrate, creatinine, homocysteine	1 [Reference]	0.89 (0.69-1.15)	0.92 (0.67-1.25)	0.53
**CHD**	1 [Reference]			
Age, treatment and energy-adjusted model	1 [Reference]	0.83 (0.69-0.99)	0.76 (0.62-0.94)	0.01
Basic model*	1 [Reference]	0.91 (0.76-1.09)	0.89 (0.72-1.11)	0.27
Basic model plus each set of risk factors below, added 1 group at a time†				
Hypertension: History of hypertension, systolic and diastolic blood pressure	1 [Reference]	0.94 (0.79-1.13)	0.94 (0.76-1.17)	0.54
Body mass index	1 [Reference]	0.93 (0.78-1.12)	0.95 (0.76-1.18)	0.57
Traditional lipids: Total, LDL[a], HDL[b] cholesterol, triglycerides (log)	1 [Reference]	0.93 (0.77-1.11)	0.95 (0.76-1.18)	0.58
Apolipoproteins: Lipoprotein(a), Apolipoprotein A1, Apolipoprotein B_{100}	1 [Reference]	0.92 (0.77-1.10)	0.91 (0.73-1.13)	0.36

© 2018 Ahmad S et al. *JAMA Network Open.*

LDL measures: LDL particle size and concentration, LDL cholesterol, Apolipoprotein B_{100}	1 [Reference]	0.92 (0.77-1.10)	0.92 (0.74-1.14)	0.52
		Mediterranean Diet		
Biomarkers	MED 0-3	MED 4-5	MED ≥ 6	P for Trend
Glucose metabolism/insulin resistances: Diabetes, hemoglobin A1c, LPIR[d], IRDRF[e], SDRF[f], DRF5[g]	1 [Reference]	0.94 (0.78-1.13)	0.94 (0.76-1.17)	0.56
Inflammation: hsCRP (log)[h], fibrinogen, ICAM-1[i], GlycA	1 [Reference]	0.93 (0.78-1.12)	0.95 (0.76-1.18)	0.57
Branched-Chain Amino Acids: total BCAA[i]	1 [Reference]	0.92 (0.77-1.10)	0.92 (0.74-1.14)	0.40
Small molecule metabolites: Citrate, creatinine, homocysteine	1 [Reference]	0.91 (0.76-1.09)	0.90 (0.72-1.12)	0.30

Age- and treatment-adjusted model are adjusted for age, treatment and energy intake (quintiles). Basic models are adjusted for age, treatment, hormonal level, postmenopausal status, current smoking, physical activity, energy intake, family history of premature MI. Participants were followed up to 12 years. LDL, Low density lipoprotein; HDL, High density lipoprotein; cLDLP, [a]LDL, Low density lipoprotein; [b]HDL, High density lipoprotein; [c]VLDL, very large density lipoproteins; [d]LPIR, Lipoprotein Insulin Resistance Index; [e]IRDRF, Insulin Resistance Diabetes Risk Factor; [f]SDRF, Short-term Diabetes Risk Factor Index; [g]DRF5, 5-Year Diabetes Risk Factor Index; [h]high sensitivity C-reactive protein; [i]sICAM-1, soluble Intercellular Adhesion Molecule 1; [j]BCAA, branched chain amino acids. P values across three levels of MED were all ≤0.05. We categorized the participants according to three levels of MED (scores of 0-3, 4-5 and 6-9).

© 2018 Ahmad S et al. *JAMA Network Open.*

eTable 2 Association of mediterranean diet with CVD events after adjustment for sets of potential mediators within total-years of follow-up (median 21.4 years)				
	Mediterranean Diet			
Biomarkers	MED 0-3	MED 4-5	MED ≥ 6	P for Trend
Age, treatment and energy-adjusted model	1 [Reference]	0.88 (0.79-0.97)	0.81 (0.71-0.91)	<0.001
Basic model*	1 [Reference]	0.94 (0.85-1.05)	0.91 (0.80-1.03)	0.12
Basic model plus each set of risk factors below, added 1 group at a time†				
Hypertension: History of hypertension, systolic and diastolic blood pressure	1 [Reference]	0.97 (0.87-1.08)	0.94 (0.83-1.07)	0.36
Body mass index	1 [Reference]	0.96 (0.86-1.07)	0.95 (0.83-1.07)	0.37
Traditional lipids: Total, LDL, HDL cholesterol, triglycerides (log)	1 [Reference]	0.96 (0.86-1.06)	0.94 (0.83-1.07)	0.35
Apolipoproteins: Lp(a), Apo A1, Apo B_{100}, Lpa (log)	1 [Reference]	0.95 (0.85-1.05)	0.92 (0.81-1.04)	0.17
LDL measures: LDL particle size and concentration, LDL cholesterol, $apoB_{100}$	1 [Reference]	0.95 (0.85-1.06)	0.93 (0.82-1.05)	0.34
HDL measure: HDL particle size and concentration, HDL cholesterol, apoA1	1 [Reference]	0.96 (0.86-1.07)	0.95 (0.83-1.07)	0.37
VLDL measures: Triglyceride-rich lipoprotein particle size and concentrations, triglycerides	1 [Reference]	0.95 (0.86-1.06)	0.94 (0.92-1.06)	0.28
Inflammation: hsCRP (log), fibrinogen, ICAM-1, GlycA	1 [Reference]	0.96 (0.86-1.07)	0.95 (0.84-1.08)	0.40
Glucose metabolism/insulin resistances: Diabetes, hemoglobin A1c, LPIR, IRDRF, SDRF, DRF5	1 [Reference]	0.97 (0.87-1.08)	0.95 (0.84-1.08)	0.42

© 2018 Ahmad S et al. *JAMA Network Open.*

Branched-Chain Amino Acids: total BCAA	1 [Reference]	0.95 (0.86-1.06)	0.93 (0.82-1.06)	0.24
Small molecule metabolites: Citrate, creatinine, homocysteine	1 [Reference]	0.94 (0.85-1.05)	0.91 (0.80-1.04)	0.15

*Basic Model included age, randomized treat assignment; smoking; menopausal status; postmenopausal hormone use; parental history of MI; exercise and total energy intake (quintiles). †Models were adjusted for the variables in the basic model plus each of the sets of risk factors added 1 group at a time to separate models. [a]LDL, Low density lipoprotein; [b]HDL, High density lipoprotein; [c]VLDL, very large density lipoproteins; [h]high sensitivity C-reactive protein; [e]sICAM-1, soluble Intercellular Adhesion Molecule 1; [f]LPIR, Lipoprotein Insulin Resistance Index; [g]IRDRF, Insulin Resistance Diabetes Risk Factor; [h]SDRF, Short-term Diabetes Risk Factor Index; [i]DRF5, 5-Year Diabetes Risk Factor Index. P values across three levels of MED were all ≤0.05. We categorized the participants according to three levels of MED (scores of 0-3, 4-5 and 6-9).

© 2018 Ahmad S et al. *JAMA Network Open.*

eTable 3 Mediation effect explained through different risk factors regarding Mediterranean diet intake with CVD events

Biomarkers	% Mediation effect explained through Counterfactual Framework	% Mediation effect explained through standard approach
Basic Model		
BMI, kg/m^2	27.0	30.8
**Blood Pressure**		
Systolic blood pressure, mmHg	28.3	25.6
Diastolic blood pressure, mmHg	16.8	15.4
**Traditional Lipids**		
LDL[a] cholesterol, mg/dL	-0.6	-2.6
HDL[b] cholesterol, mg/dL	25.1	25.6
Triglycerides, mg/dL	18.7	20.5
Total cholesterol, mg/dL	-3.6	-5.1
**Apolipoproteins**		
Lipoprotein(a), mg/dL	-1.9	-2.6
Apolipoprotein A1, mg/dL	12.1	10.3
Apolipoprotein B-100, mg/dL	5.9	5.1
**Glucose metabolism/Insulin resistance**		
Hemoglobin A1c, %	21.9	10.3
LPIR[c] (Score 1-100)	30.6	33.3
IRDRF[d] (Score 1-100)	39.4	46.2
SDRF[e] (Score 1-100)	11.7	12.8
DRF5[f] (Score 1-100)	40.8	46.2
**Inflammation pathway**		
High-sensitivity CRP, mg/L	25.3	28.2
Fibrinogen, mg/dL	10.2	7.7
Soluble ICAM-1, ng/mL	12.7	15.4
**LDL particles and size**		
LDL particle concentration, nmol/L	4.2	5.1
LDL size, nm	10.6	12.8
**HDL particles and size**		
HDL particle concentration, µmol/L	2.3	2.6
HDL size, nm	17.9	17.9
**VLDL particle and size**		
TRL[g] particle concentration, nmol/L	9.1	10.3
TRL size, nm	18.6	23.1
**Branched-Chain Amino Acids (BCAA)**		
Total BCAA, µmol/L	15.7	17.9
**Small molecule metabolites**		
Citrate, µmol/L	1.2	2.6
Creatinine, mg/dL	0.1	2.6
Homocysteine, umol/L	3.5	7.7

*Basic Model included age, randomized treatment assignment; energy intake (quintiles), smoking, menopausal status, postmenopausal hormone use, physical activity, parental history of MI before age 60. Participants were followed up to 12 years). [a]LDL, Low density lipoprotein; [b]HDL, High density lipoprotein; [c]LPIR, Lipoprotein Insulin

© 2018 Ahmad S et al. *JAMA Network Open.*

Resistance Index; [d]IRDRF, Insulin Resistance Diabetes Risk Factor; [e]SDRF, Short-term Diabetes Risk Factor Index; [f]DRF5, 5-Year Diabetes Risk Factor Index; TRL, [g]triglyceride-rich lipoproteins. P values across three levels of MED were all ≤0.05. We categorized the participants according to three levels of MED (scores of 0-3, 4-5 and 6-9).

© 2018 Ahmad S et al. *JAMA Network Open.*
